# Decoding the role of macrophage LAP3 in lung cancer – integration of single-cell technologies and machine learning reveals an orchestrating immunometabolic circuit at the tumor-epithelial interface

**DOI:** 10.3389/fimmu.2026.1749190

**Published:** 2026-02-27

**Authors:** Yunlong Dong, Xibin Fei, Mengmeng Jiang, Hongsheng Guo, Wencheng Zhang, Xu Chang, Xuanguang Li, Hongjie Zhao, Guangshun Wang

**Affiliations:** 1 Department of Thoracic Surgery, Tianjin Baodi Hospital, Baodi Hospital Affiliated to Tianjin Medical University, Tianjin, China; 2 Department of Medical Oncology, Central Hospital, Tianjin University/Third Central Hospital, Tianjin, China; 3 Department of Interventional Therapy II, Shandong Cancer Hospital and Institute, Shandong First Medical University and Shandong Academy of Medical Sciences, Jinan, Shandong, China; 4 Department of Lung Cancer Surgery, Tianjin Medical University General Hospital, Tianjin, China

**Keywords:** amino-acid metabolism, cancer immune, immunometabolism, LAP3, ligand–receptor, macrophage, NSCLC, single-cell

## Abstract

**Background:**

Amino acid metabolism plays a crucial role in shaping tumor–immune crosstalk in non–small cell lung cancer (NSCLC). However, the key cellular mediators that translate metabolic states into intercellular signals remain poorly defined.

**Methods:**

We integrated single-cell RNA-seq with spatial transcriptomics to map immunometabolic architecture. Transcriptional co-variation was decomposed into amino-acid metabolic programs using Non-negative Matrix Factorization (NMF); spatial deconvolution localized programs and cell types in tissue. Myeloid populations were subclustered to resolve macrophage states. Functional assays tested LAP3 overexpression (OE-LAP3) in A549/PC9 cells (qRT-PCR, Western blot, CCK-8, colony formation, wound-healing, Transwell) and a nude-mouse subcutaneous mouse model.

**Results:**

Integrative single-cell and spatial transcriptomic analyses revealed that tumor epithelial and myeloid cells dominate the NSCLC microenvironment and exhibit lineage-specific activation of amino acid metabolic programs. Notably, LAP3 was selectively enriched in both tumor epithelium and a distinct macrophage subset. Spatial mapping localized this LAP3-high macrophage state to epithelial–myeloid interfaces, where it functions as a signaling hub, actively secreting chemokines, cytokines, adhesion molecules, and extracellular matrix (ECM) components. To test whether LAP3 plays a causal role in tumor behavior, we established stable LAP3-overexpressing A549 and PC9 cell lines, confirming robust upregulation at both mRNA and protein levels. Functionally, LAP3 overexpression significantly suppressed proliferation—evident in CCK-8 time-course and colony formation assays—and impaired motility and invasiveness, as shown by delayed wound healing and reduced cell migration/invasion in Transwell assays. Most importantly, these effects translated *in vivo*: LAP3-overexpressing xenografts formed markedly smaller tumors in nude mice.

**Conclusions:**

LAP3 appears to functionally link amino acid catabolism to immune communication in NSCLC, defining an epithelial–macrophage immunometabolic niche where metabolic activity may shape the immune contexture. Its overexpression is associated with attenuated malignant phenotypes and heightened immune engagement, suggesting a potential dual role in restraining tumor aggressiveness and fostering an immune-responsive microenvironment. While these findings support LAP3 as a candidate biomarker for patient stratification and provide a rationale for combining metabolic modulation with immunotherapy, further mechanistic and clinical validation remains necessary.

## 1 Introduction

Non–small cell lung cancer (NSCLC) remains a leading cause of cancer-related mortality worldwide (1, 2). Although targeted therapies and immune checkpoint inhibitors have transformed outcomes for select patients, their overall efficacy is constrained by profound inter- and intra-tumoral heterogeneity. This underscores the urgent need for interpretable, clinically actionable biomarkers that capture the dynamic immunometabolic states shaping therapeutic response (3, 4).

Among the metabolic hallmarks of cancer, amino acid metabolism plays a central role—not only in fueling biomass synthesis and maintaining redox and proteostatic balance, but also in actively rewiring the tumor microenvironment (TME). Key pathways such as tryptophan–kynurenine, arginine–nitric oxide, and glutamine–glutamate serve as critical conduits through which metabolic activity modulates immune cell function and therapy sensitivity (5–8).

In this context, tumor-associated macrophages (TAMs) emerge as pivotal orchestrators of the NSCLC TME (9, 10). The lung harbors abundant resident macrophage populations—including alveolar and interstitial/monocyte-derived subsets—which, upon neoplastic transformation, exhibit remarkable plasticity along a functional continuum. TAMs coordinate diverse processes such as phagocytosis, antigen presentation, cytokine secretion, angiogenesis, and extracellular matrix remodeling (5, 11). Critically, their phenotypic and functional states are directly governed by local amino acid availability: The arginine axis balances iNOS- versus ARG1-mediated metabolism, thereby influencing T-cell effector responses; Tryptophan catabolism via IDO/TDO–kynurenine–AhR signaling drives potent immunosuppression; Glutamine, serine/glycine, and branched-chain amino acids fine-tune mTORC1 activity and dictate macrophage polarization toward inflammatory or reparative programs (12–14). Thus, TAMs function not only as metabolic sinks or sources but also as “translators” that convert biochemical cues into ligand–receptor communication, ultimately sculpting the immune landscape (15–17).

LAP3 (Leucine Aminopeptidase 3), also known as RNPEP or aminopeptidase B, belongs to the M17 family of zinc-dependent metalloproteases and functions as a metabolic enzyme that catalyzes the hydrolysis of N-terminal neutral or basic amino acids—particularly leucine—from peptides and proteins. It is implicated in antigen processing, peptide hormone maturation, cell cycle regulation, and amino acid homeostasis. Emerging evidence suggests that LAP3 is dysregulated across multiple cancers and may influence tumor progression by modulating the local availability of bioactive peptides and amino acids, thereby affecting cancer cell behavior and immune interactions. Nevertheless, its expression pattern, cellular origins, and specific role in mediating metabolism–immunity crosstalk in lung cancer remain poorly defined.

Here, we integrate single-cell and spatial transcriptomics to construct a cell-resolved, spatially anchored atlas of amino acid metabolism in NSCLC. We quantify intercellular communication across major cellular compartments and distill stable metabolic programs using matrix factorization. Within this framework, we identify LAP3—a molecule linked to peptide turnover and amino acid replenishment—as a putative regulatory hub at the epithelial–macrophage interface. LAP3 is enriched in both tumor epithelium and myeloid-rich regions and co-varies with chemokine networks, adhesion/integrin interactions, antigen-presentation machinery, and immune checkpoint modules. Cross-cohort analyses further associate LAP3 expression with features of immune infiltration and patient survival, capturing a quantifiable state of immunometabolic coupling (18, 19).

Collectively, our work delineates a mechanistic axis that originates in amino acid metabolic reprogramming, propagates through TAM-centered communication networks, and culminates in clinically relevant outcomes. This provides an interpretable, transferable framework—and identifies LAP3 as a candidate node—for guiding metabolism-immune stratification and advancing personalized therapeutic strategies in NSCLC.

## 2 Materials and methods

### 2.1 Single-cell preprocessing and integration

Analyses were performed in R (≥4.3) using Seurat (v4) and custom code. Quality control retained cells with 200–6,000 genes, and mitochondrial RNA < 10%; embryonic-biased cells were excluded. Data were normalized via SCTransform, integrated across batches with Harmony or Seurat’s integration workflow, and visualized by UMAP or t-SNE (20, 21).

### 2.2 Derivation and scoring of amino-acid metabolic programs

Amino-acid–related gene sets (KEGG/GO) and data-driven features were decomposed by NMF to derive metabolic programs (MPs). Optimal k was chosen by silhouette, consensus stability, and reconstruction error. Program scores were computed with AddModuleScore/AUCell/GSVA (ssGSEA) and compared across lineages/states.

### 2.3 Cell–cell communication

Communication was inferred using CellChat or CellPhoneDB with curated ligand–receptor databases. Pairs required ≥10% cell positivity and sufficient mean expression. Global networks and pathway-level views were generated and decomposed into incoming/outgoing heatmaps. Graph-theory centralities identified hubs. Myeloid subclusters were profiled separately (22).

### 2.4 Spatial transcriptome analysis

Spatial transcriptomic data were processed using the Seurat (v5.0) and RCTD (v1.2.0) packages in R. Quality control at the spot level was performed by filtering out spots with low total gene counts or an excessively high proportion of mitochondrial reads. Data normalization was carried out using either the SCTransform method or the NormalizeData function, depending on the downstream analysis requirements.

To infer cellular composition within each spatial spot, we performed deconvolution using RNA-seq for Cell Type Decomposition (RCTD, v1.2.0), which leverages a matched single-cell RNA sequencing (scRNA-seq) reference to map cell-type proportions onto spatial coordinates. Spatial expression patterns of LAP3, pathway activity scores, and other genes of interest were visualized using SpatialFeaturePlot. Furthermore, spatial co-localization between specific cell types or gene programs was quantitatively assessed using established metrics (23, 24).

### 2.5 Immune features and checkpoint landscape

At single-cell resolution, expression and positivity of immune checkpoints (PDCD1, CD274, CTLA4, LAG3, TIGIT), antigen presentation molecules (HLA-I/II, B2M), and chemokines/receptors were quantified. In bulk cohorts, immune infiltration (estimated by ssGSEA, xCell, or EPIC) was correlated with LAP3 expression using Spearman’s rank correlation with FDR correction.

### 2.6 Survival analyses and statistics

Kaplan–Meier curves and log-rank tests compared groups. Cox proportional hazards modeled associations with PH tested via Schoenfeld residuals. Time-dependent AUCs used timeROC. Unless stated, tests were two-sided with FDR<0.05. Random seeds were fixed; parameter lists and sessionInfo are provided in **Supplementary Materials** (25–29).

### 2.7 Cell and animal experimental

Cell lines and culture: A549 and PC9 were cultured in DMEM or RPMI-1640 (Gibco) with 10% FBS and 1% penicillin/streptomycin at 37 °C, 5% CO_2_; logarithmic-phase cells were used.

### 2.8 Wound-healing assay

A549 and PC9 cells were grouped as:①OE-NC, OE-LAP3. It onto the bottom of a 6-well plate and mark scratch lines to create wounds. Place the plate in a 37 °C, 5% CO2 cell culture incubator. At specified time points such as 0 hours and 48 hours, remove the cells from the plate and observe the width of the scratch at the same position under a microscope, taking pictures. Finally, use ImageJ software to analyze the distance of cell migration and the area of the scratch.

### 2.9 Colony-formation assay

Cells (500–1,000/well) were plated in 6-well plates and cultured for 10–14 days. Colonies were fixed (4% PFA, 15 min), stained (0.5% crystal violet, 20 min), imaged, and counted (>1 mm diameter or >50 cells). For dye quantification, stain was eluted with 10% acetic acid and read at 590 nm.

#### 2.9.1 CCK-8 proliferation assay

Cells were seeded at 2–5×10³/well in 96-well plates (edge wells filled with PBS). At 0/24/48/72/96 h, 10 μL CCK-8 was added for 1–2 h at 37 °C and absorbance was read at 450 nm with background subtraction. Curves were normalized to 0 h. ≥3 biological replicates with ≥3 technical replicates.

#### 2.9.2 OE subcutaneous mouse model

We used 6–8-week-old female BALB/c nude mice (n = 6 per group) for the xenograft experiments. Stable LAP3-overexpressing (OE-LAP3) and vector control cell lines (viability >90%) were resuspended in a 1:1 mixture of PBS and Matrigel, and 3–5 × 10^6^ cells in 100 μL were injected subcutaneously into the flanks of mice. Mice were randomized after tumor cell implantation. Tumor dimensions were measured 2–3 times per week using digital calipers, and tumor volume was calculated as (length × width²)/2. The study endpoint was defined as either a maximum tumor volume of 1,500 mm³ or day 28 post-injection, whichever occurred first.

#### 2.9.3 Statistics for functional assays

Unless otherwise specified, functional assays included ≥3 independent biological replicates, and data are presented as mean ± SD. Two-group comparisons were performed using two-tailed Student’s t-tests; multi-group comparisons used one-way ANOVA with appropriate *post hoc* tests. Statistical significance was defined as |log_2_(fold change)| > 1 and P < 0.05.

## 3 Result

### 3.1 The tumor ecosystem is dominated by an epithelium−myeloid axis with elevated amino acid metabolism

**Figure 1:**
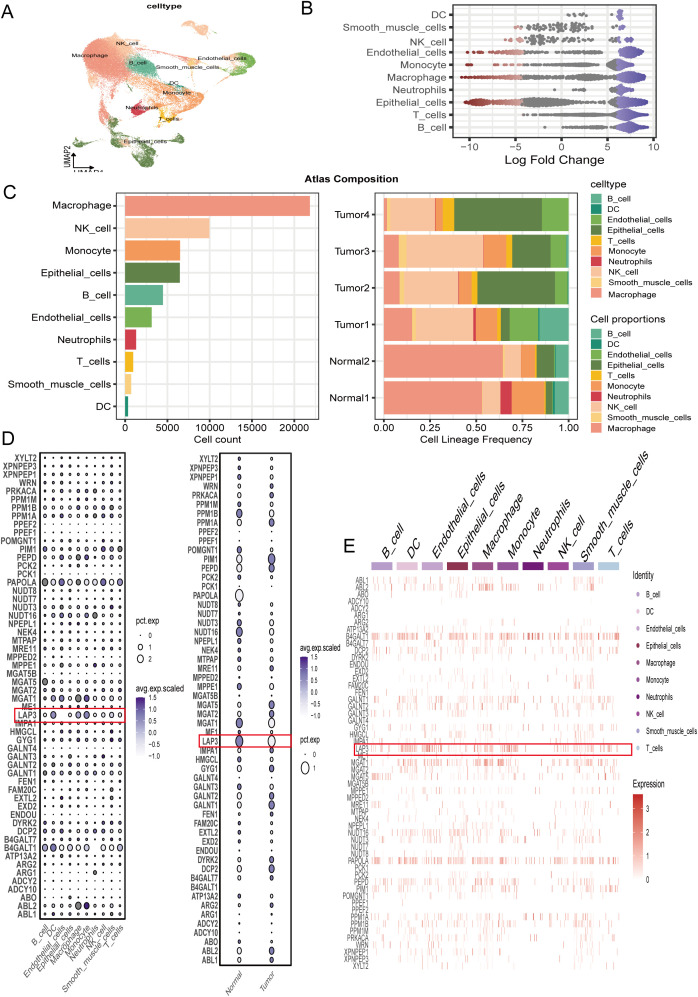
Single-cell atlas of lung cancer highlighting amino-acid–metabolism features. **(A)** UMAP displays key lung cancer cell types **(B)** Differential-expression summary showing marker strength per lineage as log fold change relative to all other cells. **(C)** Atlas composition: left, absolute cell counts by lineage; right, stacked bars showing lineage frequencies within each sample (Normal1–2, Tumor1–4). **(D)** Dot plot of amino-acid–metabolism and related genes across lineages (left) and in tumor versus normal tissues (right); dot size indicates the percentage of expressing cells (pct.exp) and color denotes scaled average expression (avg.exp.scaled). LAP3 is highlighted (red boxes). **(E)** Heat map of the same gene set across lineages (row-wise z-scores of log-normalized expression), with LAP3 highlighted. Statistics were computed with Wilcoxon rank-sum tests on single-cell data and Benjamini–Hochberg FDR correction (FDR < 0.05). DC, dendritic cell; NK, natural killer cell; UMAP, Uniform Manifold Approximation and Projection; FDR, false discovery rate.

The single-cell landscape reveals that tumor-associated epithelial and myeloid compartments (chiefly macrophages/monocytes) dominate the atlas, followed by endothelial and smooth-muscle–like cells, whereas lymphoid lineages (T, NK, B) are comparatively diffuse, jointly outlining the tumor’s cellular ecology (**Figure 1A**). In parallel, amino-acid–metabolism scores vary by lineage, being consistently higher in epithelial and myeloid cells and only modestly elevated in lymphoid subsets, indicating lineage-biased metabolic activity (**Figure 1B**). At the sample level, tumors display an enrichment of epithelial/myeloid fractions and a relative contraction of selected effector lymphocytes compared with normals, thereby shifting the system toward an “epithelium–myeloid axis” that mirrors the metabolic upregulation (**Figure 1C**). Dot plots reveal that LAP3 is preferentially expressed in dendritic cells, macrophages, and other monocycle lineages under normal conditions, but its expression is markedly reduced in tumor tissues (**Figures 1D, E**).

### 3.2 Cell–cell communication landscape coupled to immunometabolic programs

**Figure 2:**
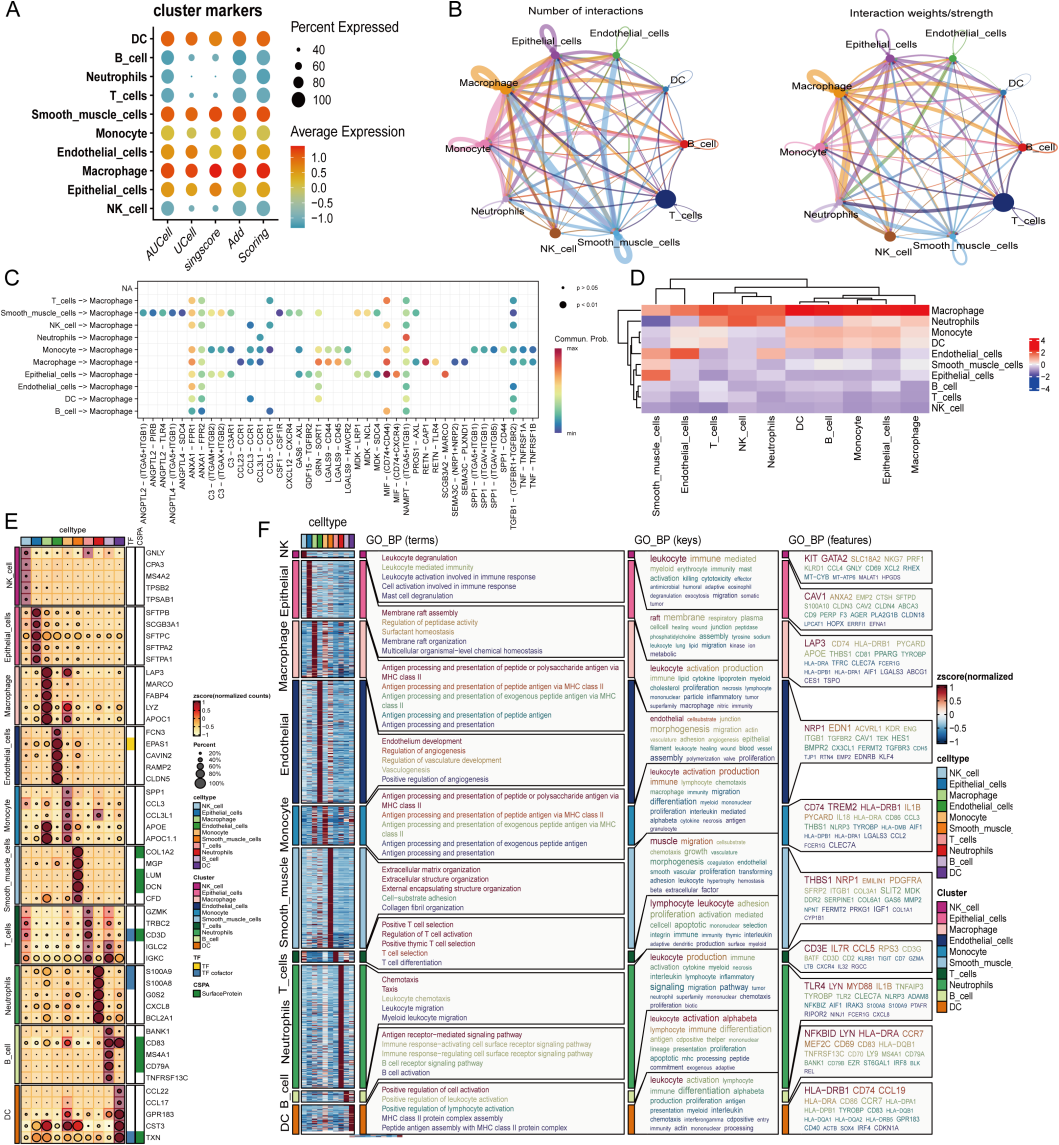
Cell–cell communication landscape and immunometabolic programs in lung cancer. **(A)** Cluster marker dot plot for the major lineages (B cell, DC, endothelial, epithelial, macrophage, monocyte, neutrophil, NK cell, smooth-muscle–like, T cell). Dot size indicates the percentage of expressing cells; color denotes scaled average expression. **(B)** Global communication networks inferred by CellChat/CellPhoneDB. Left, number of interactions between lineages; right, interaction weights/strengths. Edge width reflects interaction counts or probabilities; node size reflects the total degree. **(C)** Pathway-level ligand–receptor analysis across sender to receiver pairs. Each dot represents a curated signaling family (e.g., CCL/CXCL, GALECTIN, ICAM/PECAM, COLLAGEN/LAMININ/THBS–integrin, MIF); dot size indicates significance (*p*<0.05), and color encodes communication probability. **(D)** Heat map summarizing lineage-level centrality (z-scored) for incoming and outgoing signals, highlighting epithelial cells and macrophages as network hubs. **(E)** Gene-level dot heat map of representative adhesion/ECM, chemokine/chemokine receptors, immune checkpoints, and antigen-presentation features across lineages; dot size indicates percent expressed and color indicates scaled mean expression. **(F)** Functional annotation of lineages: left, z-score–normalized GO Biological Process (GO_BP) enrichment profiles; middle, key GO_BP terms per lineage (e.g., leukocyte activation, antigen processing and presentation, muscle migration/organization, angiogenesis, chemotaxis); right, representative feature genes supporting each term set. DC, dendritic cell; NK, natural killer cell; ECM, extracellular matrix; GO_BP, Gene Ontology Biological Process.

Building on the observed cellular compositional and transcriptional changes, we next interrogated the intercellular communication network with a specific focus on amino acid–related immunometabolic programs. Initial validation of cell identities was confirmed through cluster-marker dot plots, which reinforced stable lineage annotations and established a reliable foundation for downstream signaling inference (**Figure 2A**). At the global network level, epithelial cells, macrophages, and endothelial cells emerged as central communication hubs, characterized by dense connectivity and high interaction weights. Notably, the epithelium–macrophage, epithelium–endothelium, and monocyte–macrophage axes exhibited particularly strong signaling activity, whereas lymphoid-derived circuits were comparatively sparse (**Figure 2B**). Dissecting the ligand–receptor interactions revealed that macrophages serve as key signaling coordinators, engaging in enriched exchanges via CCL/CXCL chemokine families, integrin- and adhesion-mediated pathways, and immune receptor networks—all of which are closely associated with metabolic stress responses and immune modulation (**Figure 2C**). This is further corroborated by lineage-level centrality heatmaps, which highlight robust bidirectional communication involving macrophages; epithelial cells act as primary signal emitters toward stromal and immune compartments, while endothelial cells function as critical relays that integrate and propagate signals across multiple cellular circuits (**Figure 2D**).

At the molecular level, enzymes and transporters involved in amino acid metabolism form tightly co-expressed modules, with pronounced enrichment in both epithelial and myeloid populations. LAP3 is consistently embedded within these functional modules, reinforcing its association with core immunometabolic processes (**Figure 2E**). Finally, gene ontology (GO) enrichment analysis delineated distinct functional programs across lineages: epithelial cells are enriched for amino acid and protein processing; myeloid cells for antigen presentation and inflammatory activation; endothelial cells for angiogenesis and extracellular matrix (ECM) remodeling; and T/NK/DC populations for immune activation and antigen presentation (**Figure 2F**). Collectively, these findings delineate an integrated epithelial–immune communication circuit—orchestrated in part through endothelial intermediaries—in which metabolic activity and intercellular signaling are tightly coupled. LAP3 resides at the intersection of this network, positioning it as a potential regulator and biomarker of this immunometabolic axis.

### 3.3 Incoming and outgoing signaling patterns

**Figure 3:**
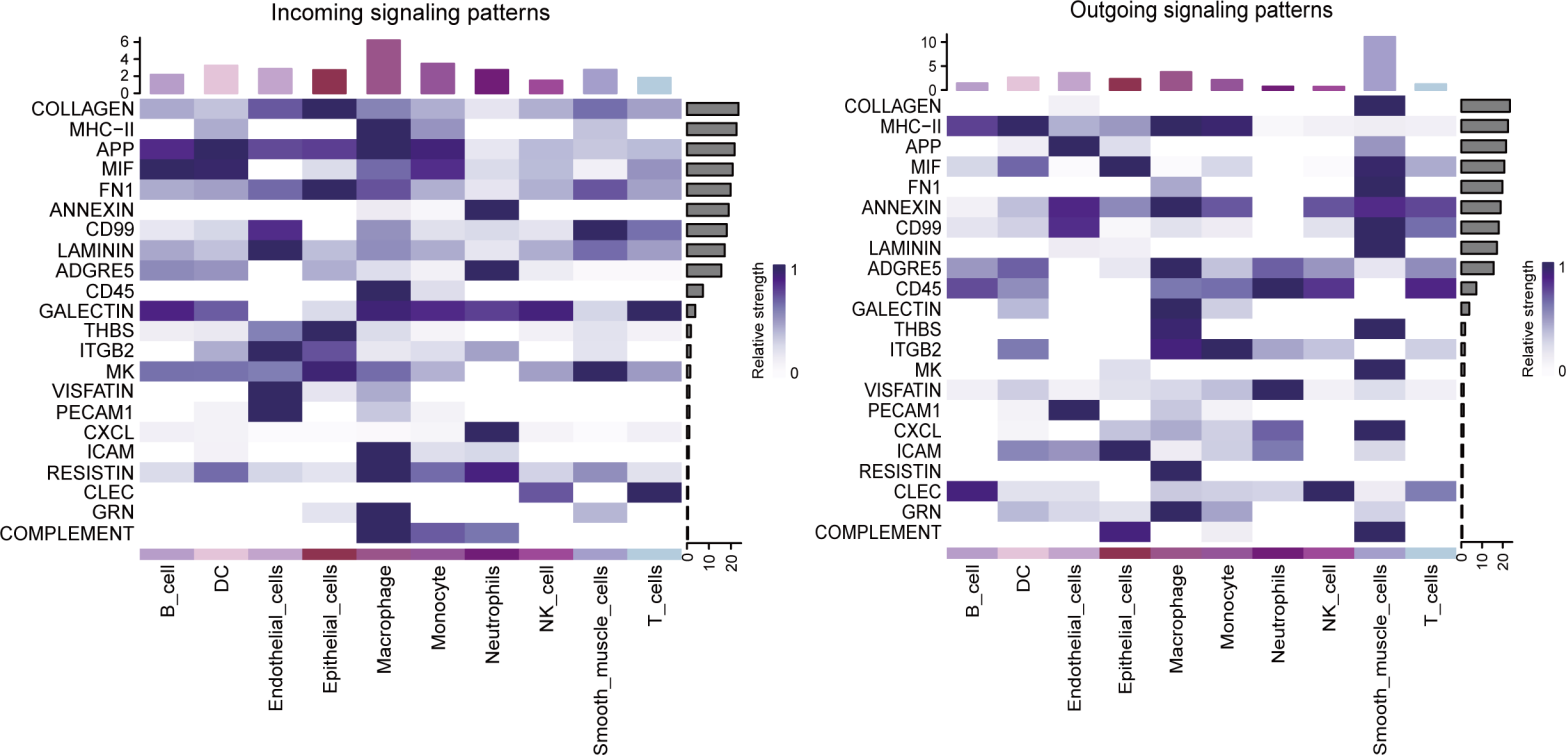
Incoming and outgoing signaling patterns across lineages in the lung-cancer single-cell atlas. Heatmaps summarize ligand–receptor communication inferred by CellChat/CellPhoneDB.

To dissect directionality, incoming vs. outgoing communication heat maps delineate pathway sources and sinks. Matrix/adhesion programs (COLLAGEN, LAMININ, FN1, THBS/integrins) are predominantly emitted by epithelial/endothelial cells and received by macrophages, monocytes, and smooth-muscle–like cells. In contrast, inflammatory/immune-modulatory routes (MIF, GALECTIN, CD45/CD99, CXCL, RESISTIN, CLEC, GRN, COMPLEMENT, MHC-II) display a myeloid “high-in/high-out” signature, underscoring their hub role. Endothelium shows strong incoming ICAM/PECAM signals, consistent with a relay function. Lymphoid bands are weaker or more selective, with notably low T-cell output, matching the constrained lymphoid communication in this cohort. These patterns align with **Figures 1**, **2**: metabolically active epithelial/myeloid cells also maintain tightly coupled interaction networks, reinforcing metabolism–immunity coupling (**Figure 3**).

### 3.4 NMF metabolic programs and spatial mapping of LAP3

**Figure 4:**
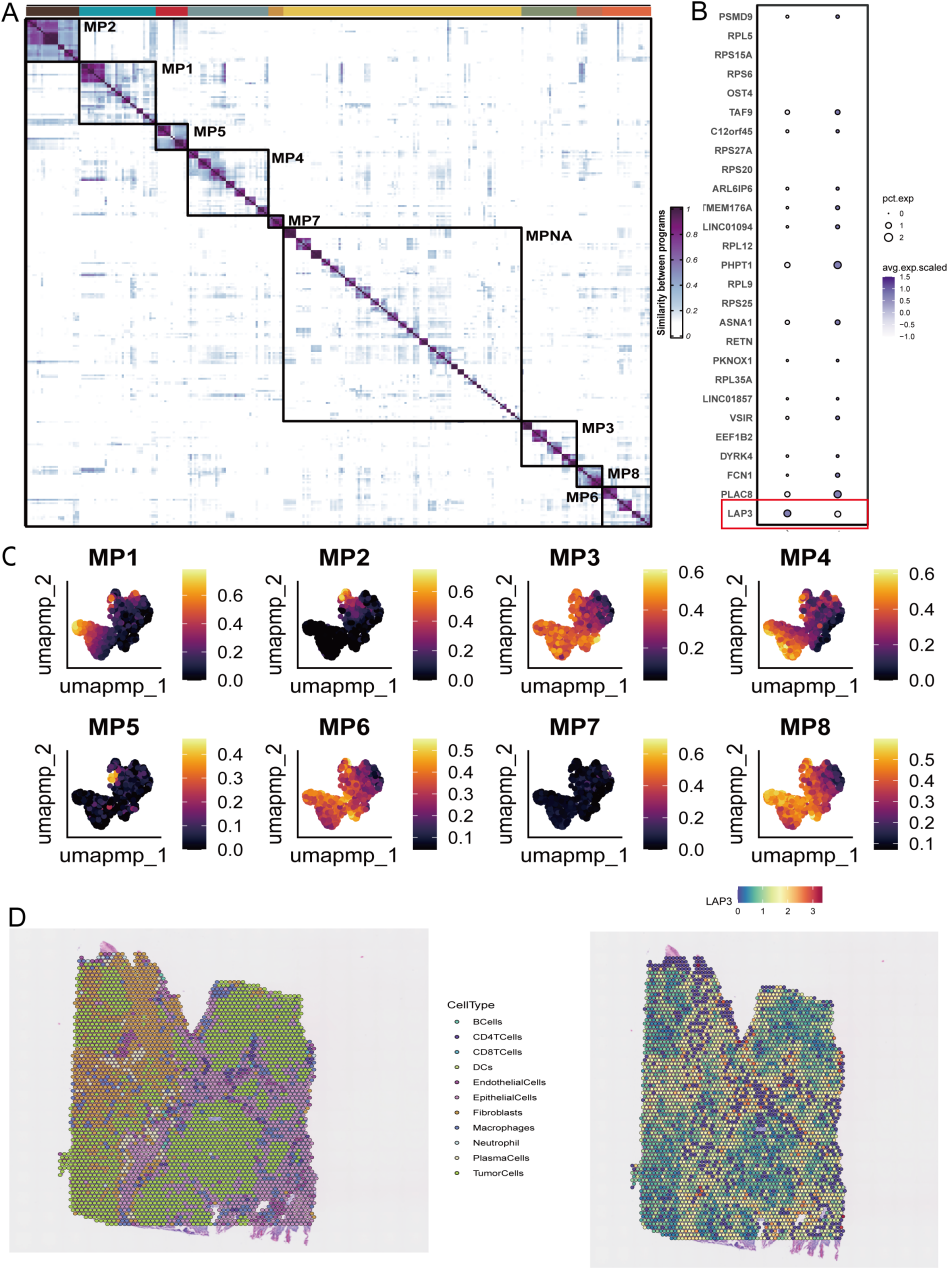
NMF-derived metabolic programs and their spatial distribution with LAP3 focus. **(A)** Program–program similarity matrix showing block-diagonal structure of eight metabolic programs (MP1–MP8). Warmer colors indicate higher similarity. A larger super-module encompasses co-varying programs. **(B)** Dot plot of representative high-loading; dot size indicates the percentage of expressing cells (pct.exp) and color denotes scaled average expression. LAP3 is highlighted (red box). **(C)** UMAPs of single cells colored by normalized scores (0–1) for each program (MP1–MP8), illustrating lineage-biased enrichment patterns. **(D)** Spatial transcriptomics of a representative section: left, spot-level cell-type deconvolution map; right, spatial distribution of LAP3 expression/score. MP, metabolic program; MPNA, meta-program; UMAP, Uniform Manifold Approximation and Projection.

To move from genes to programs, we decomposed transcriptional co-variation into discrete amino-acid metabolic programs. The similarity matrix exhibits a clear block-diagonal architecture, separating MP1–MP8 from a diffuse background, thereby indicating cohesive yet distinct programs (**Figure 4A**). A representative gene dot plot shows that these programs comprise enzymes and transporters; notably, LAP3 clusters with PLAC8 within the same feature set and is more highly expressed, supporting its role as a core marker of this axis (**Figure 4B**). When scores are projected onto the single-cell embedding, program topographies emerge—some concentrated in tumor epithelium and others in myeloid cells—suggesting a division of metabolic labor across lineages (**Figure 4C**). Spatial transcriptomics then anchors these signals: deconvolution maps juxtaposed belts of tumor cells and macrophages, while LAP3 peaks in tumor-rich regions and at interfaces abutting myeloid zones, consistent with a metabolic–immune intersection (**Figure 4D**). In sum, these analyses translate reprogramming into reusable metabolic programs and spatially align them with epithelial–myeloid interactions, again highlighting LAP3 as a hub.

### 3.5 Macrophage metabolic heterogeneity and LAP3-high hub subcluster

**Figure 5:**
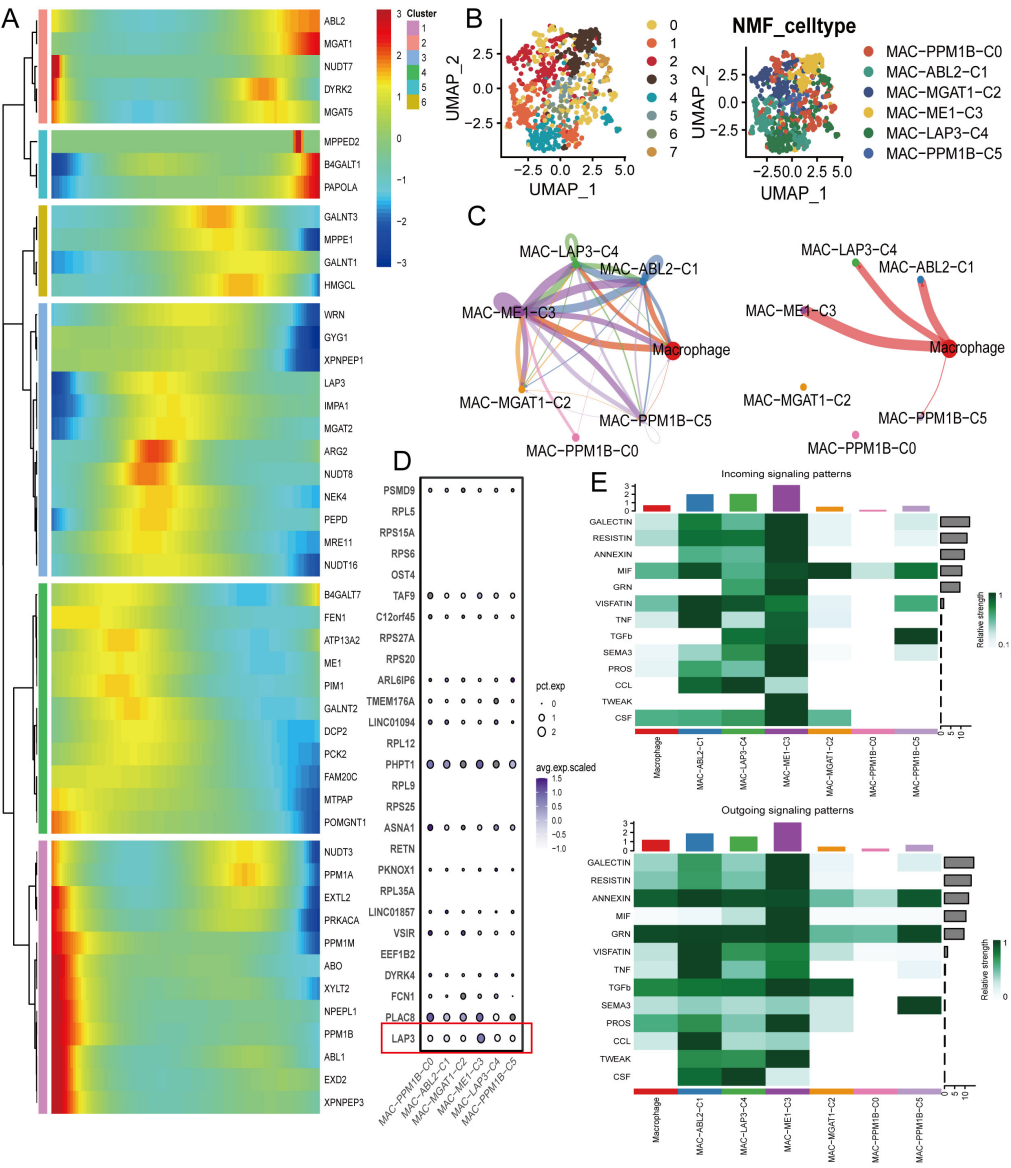
Macrophage-centered immunometabolic states and communication signatures. **(A)** Z-score heat map of amino-acid–metabolism/proteostasis genes across macrophage subclusters derived by NMF; rows, genes; columns, cluster-averaged expression (cluster IDs at the top). **(B)** UMAP of the myeloid compartment showing Leiden clusters (left) and NMF-defined macrophage states (right): MAC-PPM1B-C0, MAC-ABL2-C1, MAC-MGAT1-C2, MAC-ME1-C3, MAC-LAP3-C4, and MAC-PPM1B-C5. **(C)** Circle networks summarizing the number of interactions (left) and interaction weights/strength (right) inferred among macrophage states; edge width encodes counts/strength and node size encodes total degree; the LAP3 state is indicated. **(D)** Dot plot of representative markers per state; dot size indicates the percentage of expressing cells (pct.exp) and color denotes scaled average expression (avg.exp.scaled); LAP3 is highlighted. **(E)** Incoming (top) and outgoing (bottom) signaling patterns for macrophage states across curated families (e.g., GALECTIN, RESISTIN, ANNEXIN, MIF, VISFATIN, TGFB, SEMA3, PROS, COLLAGEN, TWEAK, CSF); right-side bar plots show cumulative strengths. NMF, non-negative matrix factorization; UMAP, Uniform Manifold Approximation and Projection; ECM, extracellular matrix.

Given the myeloid centrality, we resolved macrophage heterogeneity along the amino-acid axis. The gene–cell heat map defines modular enzyme/transport patterns and delineates NMF-derived subclusters (**Figure 5A**). In low-dimensional space, these subclusters separate cleanly; the MAC-LAP3-C4 (LAP3-high) group occupies a distinct territory relative to ME1-, MGAT1-, and PPM1B- states, indicating metabolic division of labor within macrophages (**Figure 5B**). Communication graphs then highlight hub-like behavior for LAP3-high cells—with high edge counts and weights to the pan-macrophage pool and to other macrophage subclusters—forming a tightly coupled myeloid subnet (**Figure 5C**). Marker plots corroborate this: LAP3 and PLAC8 are co-upregulated in MAC-LAP3-C4 alongside ribosomal/protein-processing features (**Figure 5D**). Finally, incoming/outgoing patterns show a myeloid high-in/high-out signature, most pronounced in MAC-LAP3-C4 across GALECTIN, RESISTIN, ANNEXIN, MIF, GRN, TNF routes, with notable CSF/chemokine outputs that position this cluster as a network organizer of stromal–immune interactions (**Figure 5E**).

### 3.6 Feature selection and model stability; LAP3 expression and prognostic stratification

**Figure 6:**
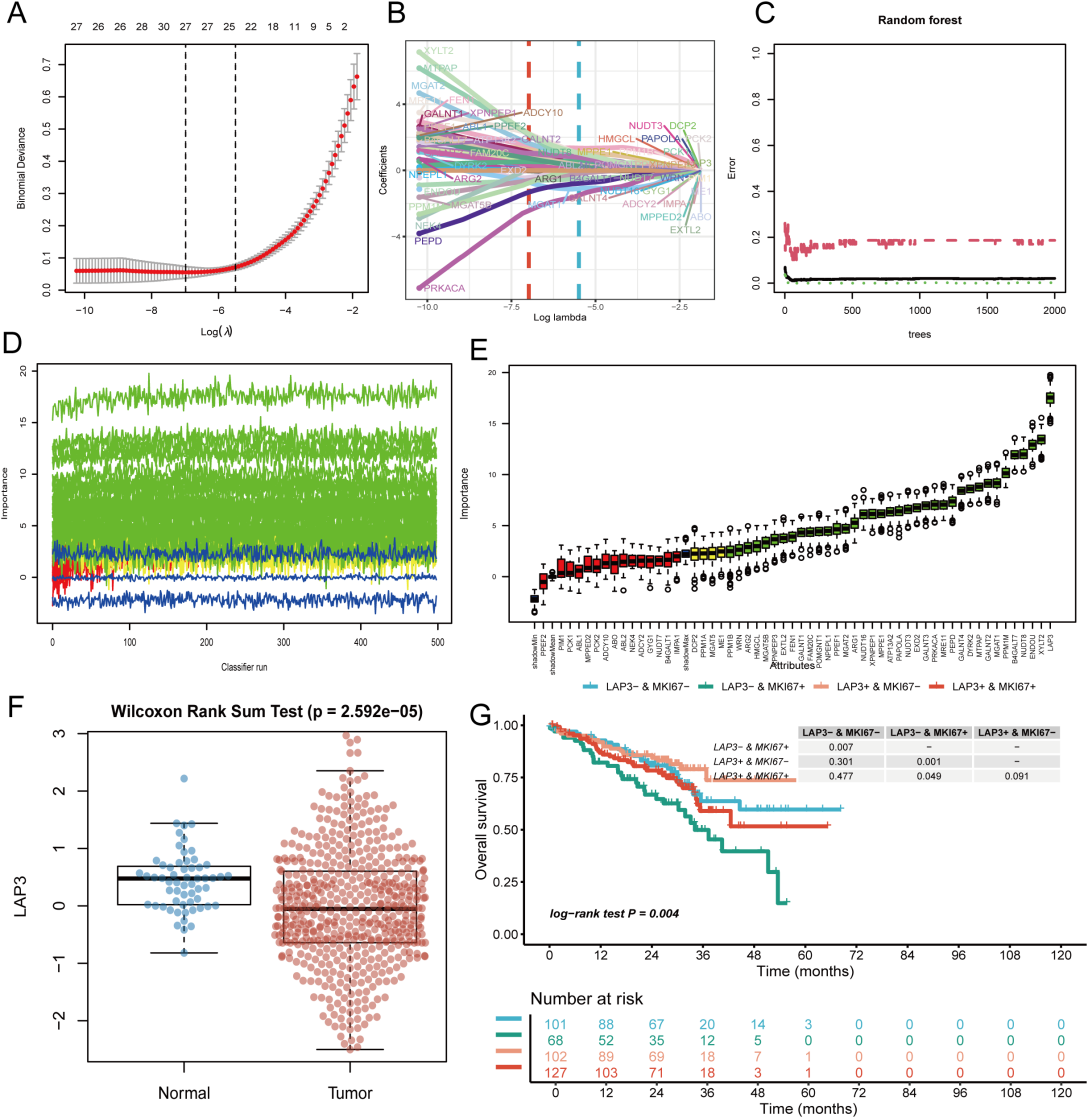
Feature selection, model tuning, and clinical relevance of LAP3. **(A)** Ten-fold cross-validation curve for the penalized regression (LASSO), showing binomial deviance across log(λ); numbers on top denote the non-zero predictors at each λ. **(B)** LASSO coefficient trajectories as a function of log(λ); dashed lines mark the selected λ values and the subset of retained features. **(C)** Random-forest out-of-bag error versus the number of trees, showing error stabilization used to choose tree depth/size. **(D)** Stability analysis of variable importance across repeated resampling/iterations (500 runs), with colored traces representing individual predictors and demonstrating consistent ranking. **(E)** Aggregated importance scores (box/whisker plots) for candidate genes/features across models and resamples; boxes summarize interquartile ranges and points denote outliers. **(F)** Differential expression of LAP3 between normal and tumor samples (box/dot plot); Wilcoxon rank-sum test p-value is shown. **(G)** Kaplan–Meier overall survival curves stratified by combined LAP3 and MKI67 status (four groups); the log-rank test indicates significant separation (p = 0.004). The table lists numbers at risk and pairwise p-values. LASSO, least absolute shrinkage and selection operator; OOB, out-of-bag; KM, Kaplan–Meier; OS, overall survival.

We next assessed model robustness and clinical relevance for LAP3. Cross-validated Least Absolute Shrinkage and Selection Operator (LASSO) identified a stable penalty window, with λ_min and λ_1SE bracketing a sparse yet discriminative solution (**Figure 6A**); coefficient paths illustrate early entry of metabolism-related features as regularization relaxes (**Figure 6B**). In parallel, random-forest error plateaued rapidly with tree number (**Figure 6C**). Repeated runs yielded stable variable-importance trajectories (**Figure 6D**) and a consistent ranking across resamples (**Figure 6E**). At the expression level, LAP3 is upregulated in tumors vs. normals (Wilcoxon P = 2.592×10^-5^; **Figure 6F**). Moreover, combined stratification by LAP3 and MKI67 produced stepwise overall-survival separation—double positive worst, double negative best, intermediates in between (log-rank P = 0.004; **Figure 6G**). Collectively, feature selection, stability analyses, differential expression, and prognosis all converge to implicate LAP3 as a clinically meaningful metabolic node.

Our analysis reveals that LAP3 is indeed dysregulated in multiple cancer types beyond lung cancer. Specifically, LAP3 expression is significantly upregulated in breast invasive carcinoma (BRCA), esophageal carcinoma (ESCA), glioblastoma multiforme (GBM), head and neck squamous cell carcinoma (HNSC), prostate adenocarcinoma (PRAD), and stomach adenocarcinoma (STAD). Conversely, LAP3 expression is markedly downregulated in cholangiocarcinoma (CHOL), kidney renal papillary cell carcinoma (KIRP), liver hepatocellular carcinoma (LIHC), lung adenocarcinoma (LUAD), lung squamous cell carcinoma (LUSC), and pheochromocytoma and paraganglioma (PCPG) (**Supplementary Figure 1A**). Furthermore, we also discovered that the mutation of LAP3 is a key factor contributing to the progression of lung cancer (**Supplementary Figure 1C**).

### 3.7 Machine learning models identify LAP3 gene signatures with high accuracy in TCGA-LUAD

**Figure 7:**
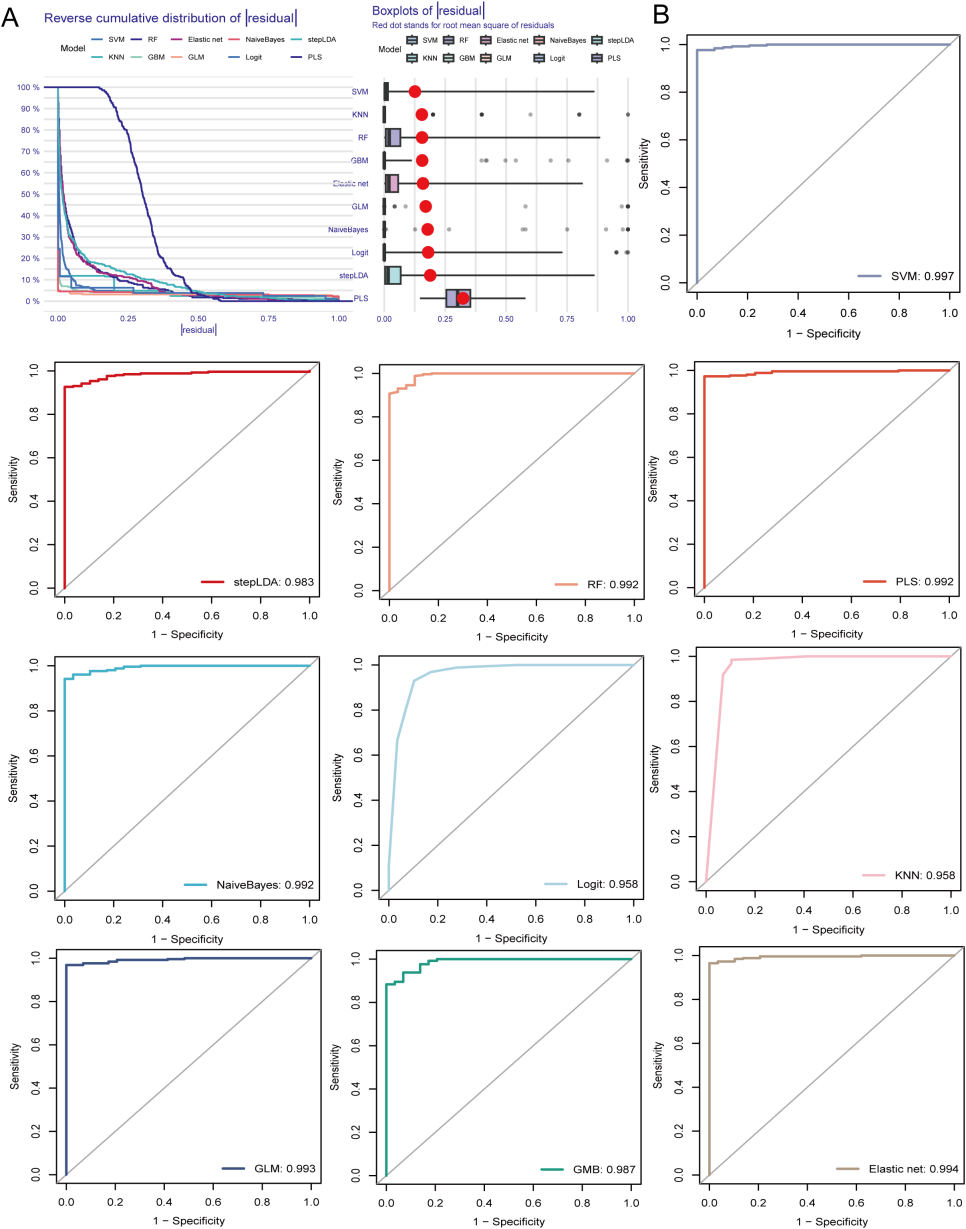
Model comparison for TACE-benefit classification using residual-based labels. **(A)** Reverse cumulative distribution (left) and boxplots (right) of absolute residuals across candidate algorithms. Curves summarize dispersion of residuals; smaller areas and tighter boxes indicate better fit. Red circles in boxplots denote the root mean square of residuals for each model. **(B)** Receiver-operating characteristic (ROC) curves for representative classifiers. Grey diagonal indicates random performance. Reported AUCs include: SVM 0.997, GLM 0.993, RF 0.992, PLS 0.992, NaiveBayes 0.992, Elastic net 0.994, stepLDA 0.983, GBM 0.987, KNN 0.958, and Logistic (Logit) 0.958, indicating uniformly strong discrimination with SVM performing best. AUC, area under the curve; GBM, gradient boosting machine; GLM, generalized linear model; KNN, k-nearest neighbors; PLS, partial least squares; RF, random forest; SVM, support vector machine; stepLDA, stepwise linear discriminant analysis.

In this study, we utilized the TCGA-LUAD (Lung Adenocarcinoma) cohort to develop and evaluate multiple machine learning models. **Figure 7A** presents a comparative analysis of model fitting accuracy through reverse cumulative distribution plots and residual boxplots. The results highlight that the Support Vector Machine (SVM) model exhibits the lowest Root Mean Square Error (RMSE) with residuals tightly clustered around zero, indicating superior predictive performance compared to other models such as PLS, Elastic net, and Random Forest. These findings suggest that SVM effectively captures the complex immune-related signals within the dataset. **Figure 7B** evaluates the classification performance of each model using Receiver Operating Characteristic (ROC) curves. All models demonstrate excellent discrimination capabilities (AUC > 0.95). Notably, the SVM model achieves the highest AUC value (0.997), significantly outperforming alternatives like Random Forest (AUC = 0.992), PLS (AUC = 0.992), and Gradient Boosting Machines (AUC = 0.987). Other models, including Logistic Regression (AUC = 0.955), K-Nearest Neighbors (AUC = 0.958), and Naive Bayes (AUC = 0.992), also show strong discriminatory power, whereas stepLDA (AUC = 0.583) performs notably worse, indicating its limitations in handling non-linear data structures. We also using an independent dataset from TCGA lung adenocarcinoma (LUAD) cohorts. Instead of relying solely on the multi-gene immune signature, we evaluated the diagnostic performance of LAP3 expression alone as a binary classifier to distinguish tumor from normal tissue. This analysis yielded an AUC of 0.886, demonstrating robust discriminatory power without overfitting (new **Supplementary Figure 1B**).But, we acknowledge certain limitations in this predictive analysis. The original high-performance models were derived from a single institutional cohort with relatively limited sample size, which increases the risk of overfitting despite cross-validation.

### 3.8 LAP3 defines an immune active yet checkpoint enriched tumor microenvironment

**Figure 8:**
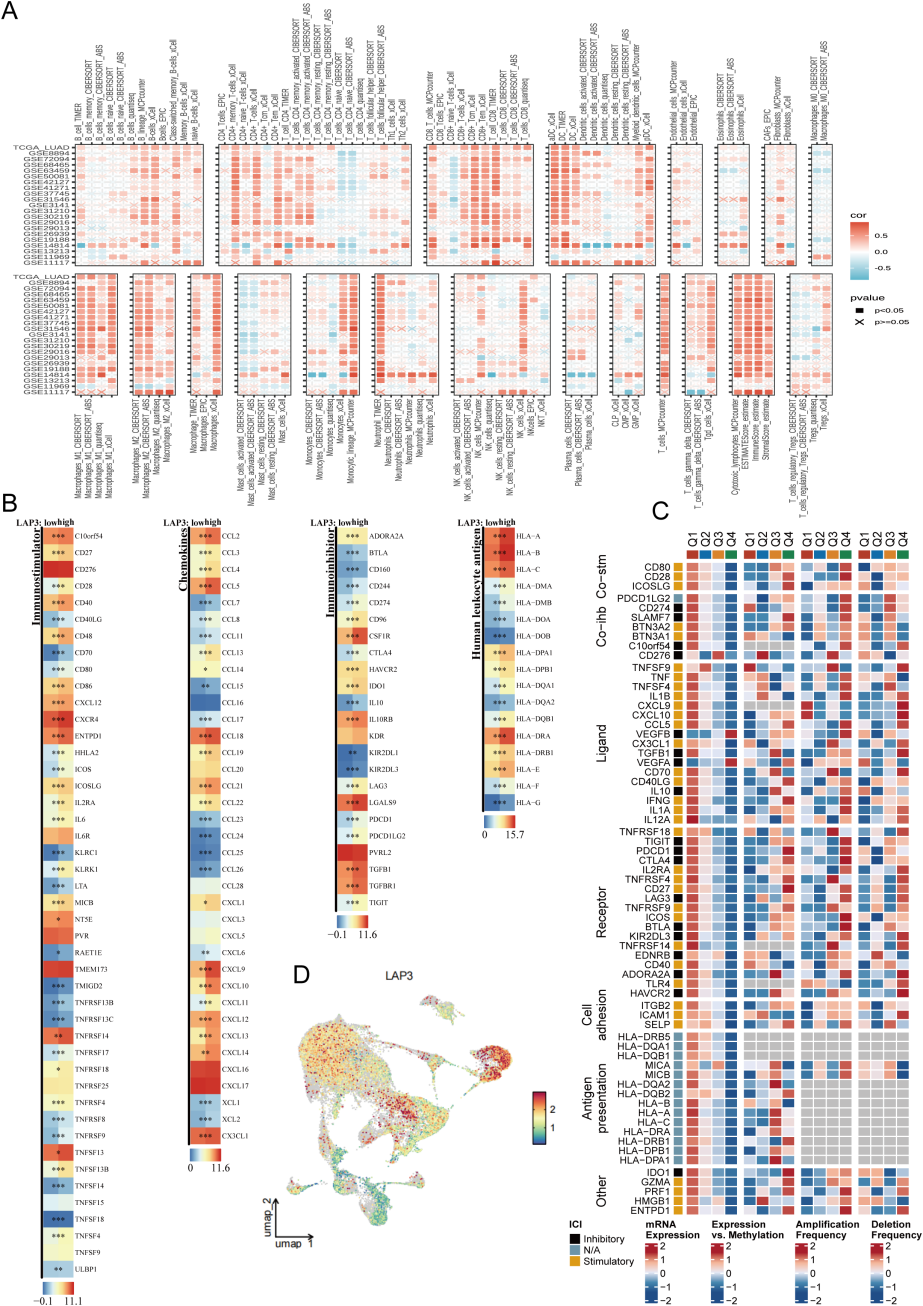
LAP3 associates with the tumor–immune landscape and checkpoint circuitry in lung cancer. **(A)** Cross-cohort correlation maps showing associations between LAP3 expression and immune features in bulk datasets. Tiles display Spearman correlation, with boxes grouping feature sets; denotes non-significant tests (FDR≥0.05). **(B)** Differential analysis between LAP3-low and LAP3-high groups for four categories—immunostimulators, chemokines, immunoinhibitors, and HLA genes. Bars/tiles indicate effect size (log fold change) and significance. **(C)** Multi-omic checkpoint panel summarizing co-stimulatory/co-inhibitory axes (ligands, receptors, adhesion and antigen-presentation modules) across LAP3 quartiles. Heatmaps show expression, expression–methylation coupling, and copy-number amplification/deletion frequencies; left annotation indicates stimulatory/inhibitory class. **(D)** Single-cell UMAP colored by LAP3 levels, illustrating its distribution across cell populations. HLA, human leukocyte antigen; ICI, immune checkpoint; ECM, extracellular matrix; UMAP, Uniform Manifold Approximation and Projection; FDR, false discovery rate.

Having established modeling robustness, we delineated LAP3’s immune context. Cross-cohort correlation heat maps show reproducible associations: LAP3 tracks positively with myeloid and epithelial programs and relates to effector lymphocyte and antigen-presentation modules, with coherent red/blue patterns and frequent significance marks (**Figure 8A**). When stratifying tumors by LAP3 levels, we observe coordinated increases in immune-inhibitory checkpoints, chemokines/receptors, stimulatory molecules, and HLA genes in LAP3-high tumors, suggesting that metabolic activation coexists with adaptive immune suppression (**Figure 8B**). A checkpoint compendium integrating mRNA, expression–methylation coupling, and copy-number alterations maps both inhibitory and stimulatory axes; LAP3-associated upregulation frequently coincides with local hypomethylation or focal gains, indicating potential genomic/epigenetic co-regulation (**Figure 8C**). Single-cell visualization places LAP3 predominantly in tumor epithelium and myeloid cells, forming gradients at their interfaces—precisely where communication hubs and metabolic programs converge (**Figure 8D**). Overall, the LAP3-high state aligns with “chemokine activation + checkpoint upregulation + enhanced antigen presentation,” offering actionable cues for immunotherapy stratification and metabolic-immune combinations.

### 3.9 LAP3 overexpression suppresses tumor proliferation, migration, and *in vivo* growth

**Figure 9:**
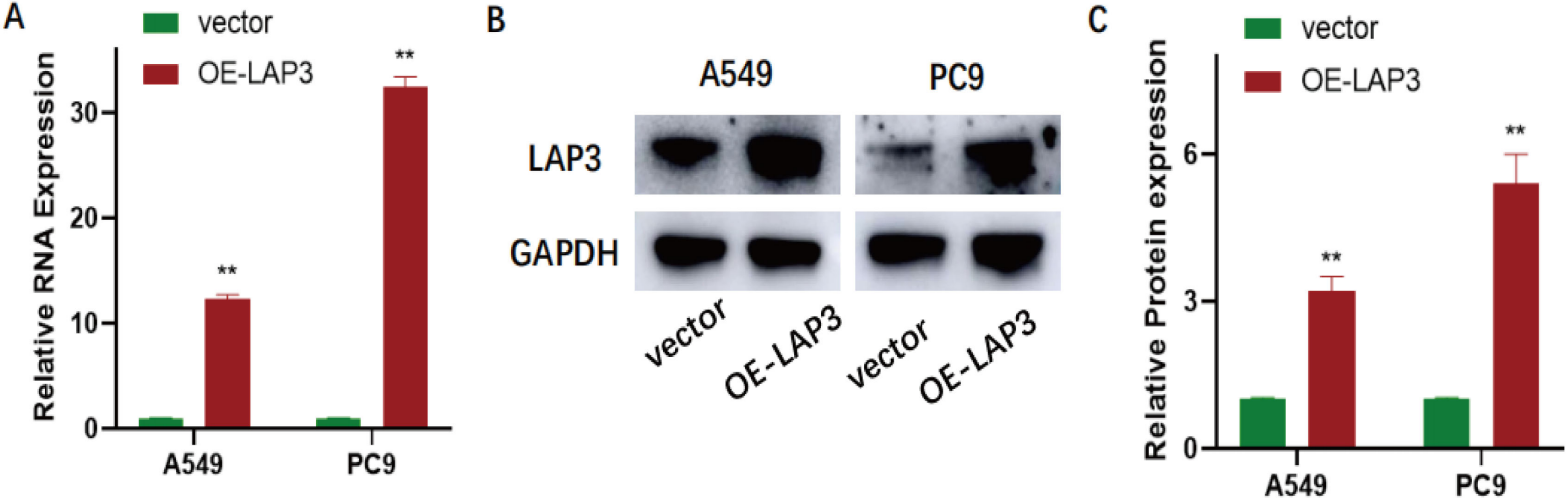
Validation of LAP3 overexpression in NSCLC cells (A549, PC9). **(A)** qRT–PCR showing markedly increased LAP3 mRNA in OE-LAP3 cells versus vector controls; values are normalized to a housekeeping gene and presented as mean ± SD (n = 3). **P < 0.01 vs. vector. **(B)** Immunoblot confirming elevated LAP3 protein in OE-LAP3 cells; GAPDH serves as the loading control. **(C)** Densitometric quantification of LAP3 protein levels normalized to GAPDH (mean ± SD, n = 3). **P < 0.01 vs. vector.

**Figure 10:**
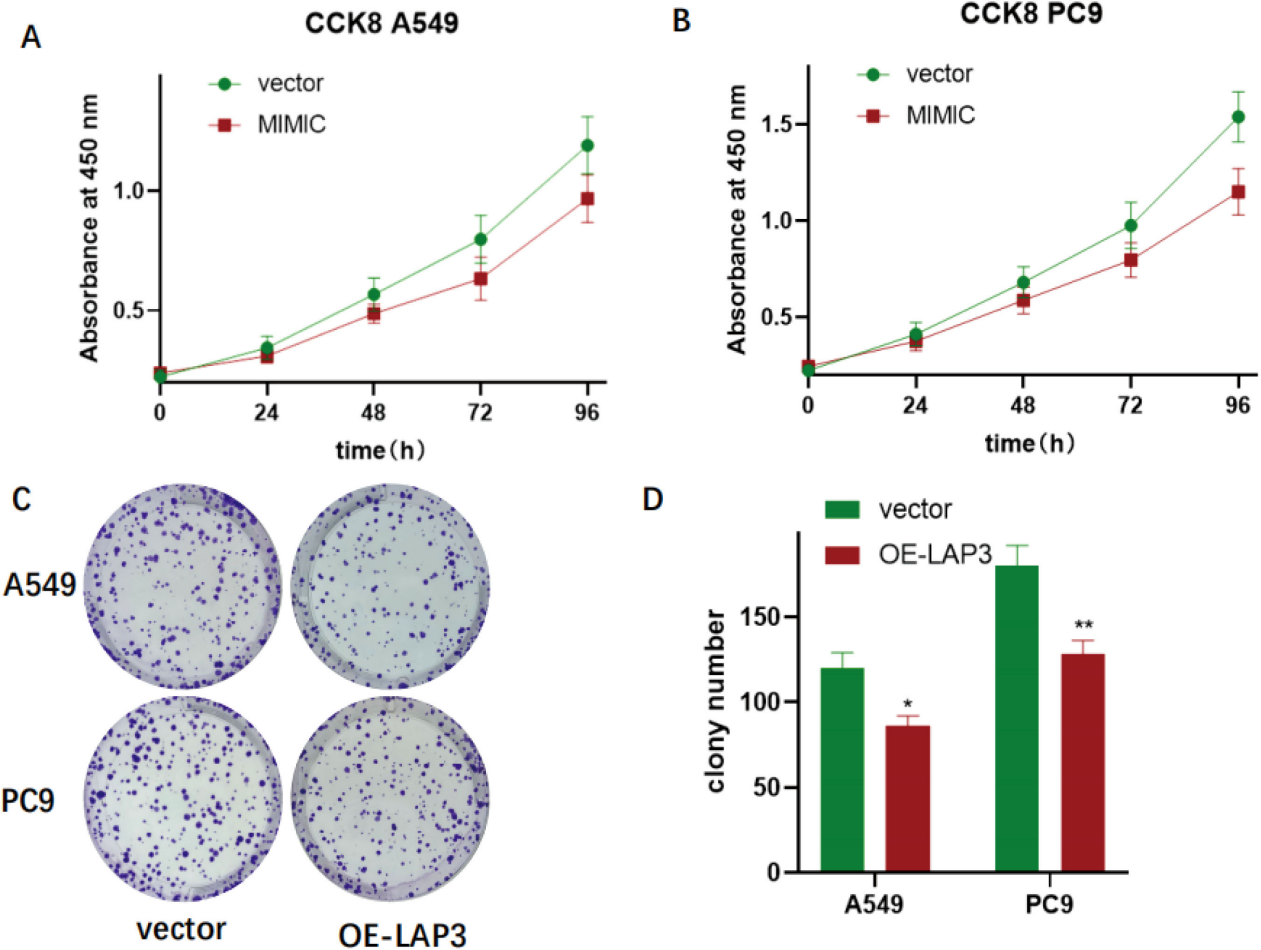
LAP3 perturbation suppresses proliferation and clonogenic growth in NSCLC cells. **(A, B)** CCK-8 time-course assays in A549 **(A)** and PC9 **(B)** comparing control (vector) with the LAP3-MIMIC group. Absorbance at 450 nm was recorded at 0, 24, 48, 72, and 96 h; LAP3-MIMIC shows reduced growth relative to vector. **(C, D)** Colony-formation assays for A549 and PC9 cells with LAP3 overexpression (OE-LAP3) versus vector. Representative crystal-violet plates **(C)** and quantification of colony numbers **(D)** indicate fewer colonies upon LAP3 overexpression. Data are mean ± SD from three independent experiments; two-tailed Student’s *t*-test. *P* < 0.05; P < 0.01 vs. Vector.

**Figure 11:**
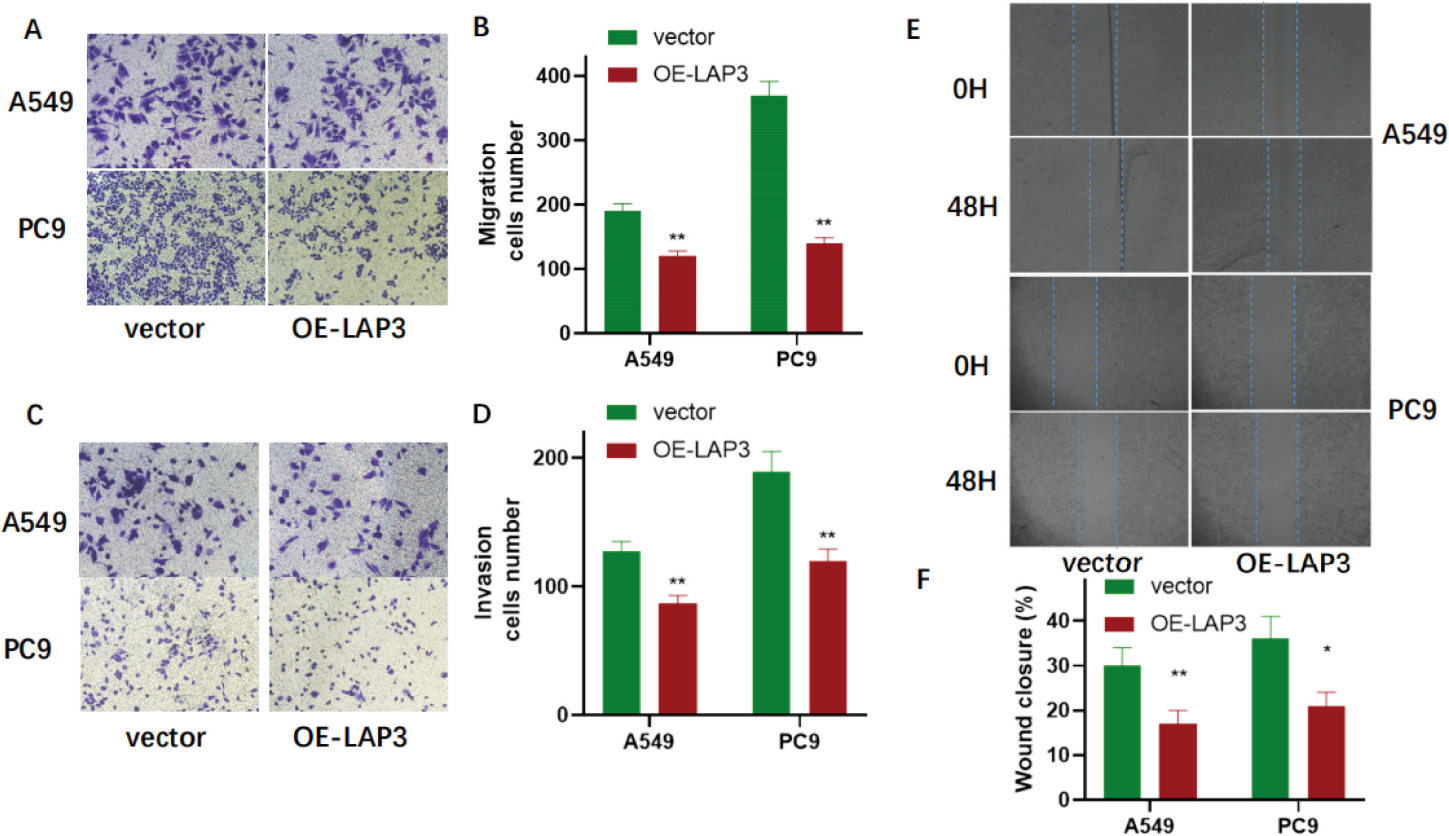
LAP3 overexpression suppresses migration, invasion, and wound healing in NSCLC cells. **(A)** Representative images of Transwell migration assays in A549 and PC9 cells transfected with control vector or OE-LAP3. Cells were stained with crystal violet after 24 h incubation. Scale bar: 100 μm. **(B)** Quantification of migrated cell numbers from **(A)**. Data represent mean ± SD from three independent experiments. **P < 0.01 vs. vector group. **(C)** Representative images of Matrigel-coated Transwell invasion assays in A549 and PC9 cells. Cells were stained with crystal violet after 48 h. Scale bar: 100 μm. **(D)** Quantification of invasive cell numbers from **(C)**. Data represent mean ± SD from three independent experiments. **P < 0.01 vs. vector group. **(E)** Representative phase-contrast images of wound-healing (scratch) assays in A549 and PC9 cells at 0 h and 48 h post-scratch. Dashed lines indicate initial wound edges. **(F)** Quantification of wound closure percentage at 48 h. Data represent mean ± SD from three independent experiments. *P < 0.05, **P < 0.01 vs. vector group.

**Figure 12:**
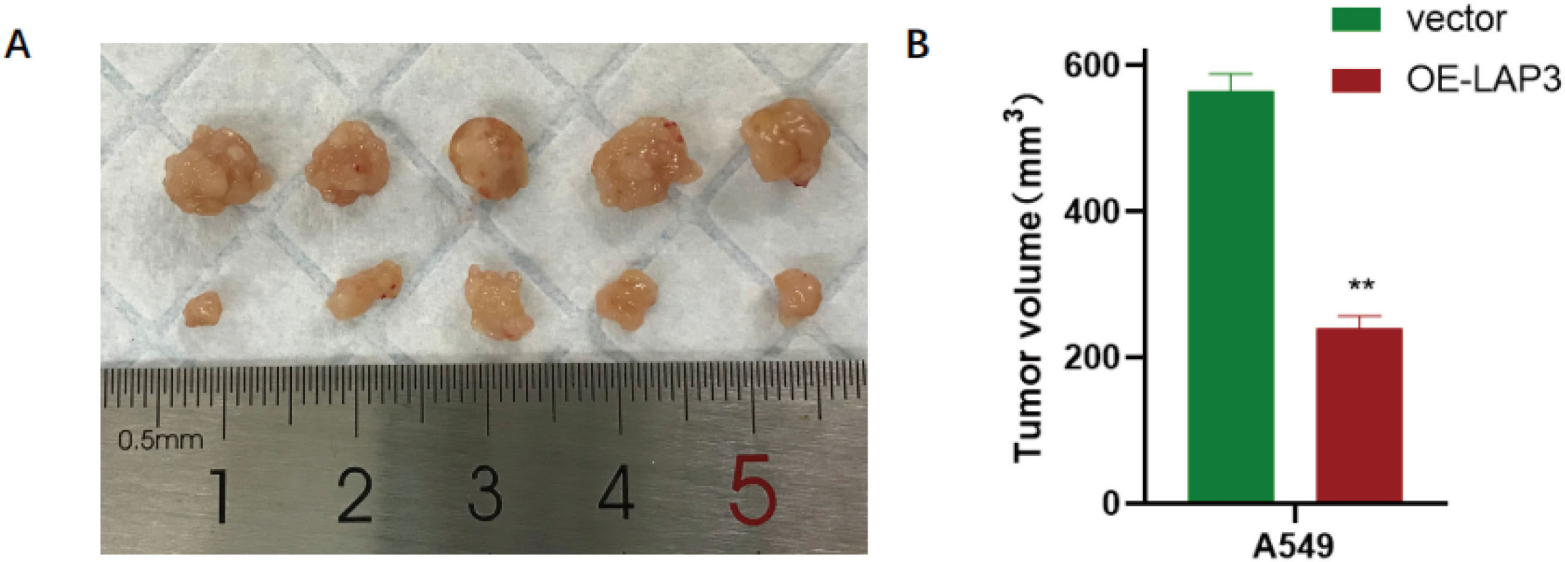
LAP3 overexpression suppresses *in-vivo* tumor growth. **(A)** Representative photographs of subcutaneous mouse harvested at endpoint from nude mice injected with A549 cells stably overexpressing LAP3 (OE-LAP3) or vector control. Ruler indicates centimeters. **(B)** Tumor volumes (mm³) at endpoint show significantly smaller tumors in the OE-LAP3 group compared with vector (P < 0.01, two-tailed Student’s *t*-test).

To functionally validate LAP3 as a key regulated gene, we established stable LAP3-overexpressing A549 and PC9 NSCLC cell lines. qRT-PCR and immunoblotting confirmed significant upregulation of LAP3 at both mRNA and protein levels compared to vector controls (P < 0.01; **Figures 9A–C**). Using these models, we assessed the phenotypic consequences of LAP3 elevation. CCK-8 proliferation assays revealed significantly reduced cell growth over 24–96 hours in both cell lines (**Figures 10A, B**), and colony formation assays demonstrated markedly decreased clonogenic potential (A549: P < 0.05; PC9: P < 0.01; **Figures 10C, D**). Furthermore, LAP3 overexpression impaired cell motility and invasiveness: wound-healing assays showed delayed closure at 48 hours (A549: P < 0.01; PC9: P < 0.05; **Figures 11E, F**), and Transwell assays confirmed fewer migrated and invaded cells (P < 0.01; **Figures 11A–D**). Critically, these *in vitro* effects translated to *in vivo* tumor suppression. In a subcutaneous xenograft mouse model, tumors derived from LAP3-overexpressing cells exhibited significantly reduced volume compared to controls (P < 0.01; **Figures 12A, B**). Collectively, these orthogonal functional experiments demonstrate that LAP3—initially identified through integrated single-cell, spatial, and computational analyses—not only serves as a biomarker but also exerts tumor-suppressive activity by dampening proliferation, migration, invasion, and *in vivo* tumor growth, thereby reinforcing its central role within the proposed metabolic-immune regulatory framework.

## 4 Discussion

This study integrates single-cell and spatial transcriptomics with communication inference and functional assays to reveal a coherent metabolism–immunity circuit in lung cancer. Tumor epithelium and myeloid compartments, especially macrophages and monocytes, form a coupled axis with elevated amino-acid metabolic programs and dense interlineage signaling. Across data layers, LAP3 repeatedly marks this interface.

Program decomposition shows that amino-acid enzymes and transporters assemble into stable modules that map to epithelial–myeloid contact zones. Within the myeloid space, a LAP3-high macrophage state behaves as a communication hub, broadcasting chemokine, cytokine, and adhesion signals and receiving strong inputs from neighboring lineages.

experiments are consistent with this architecture. Enforced LAP3 expression reduces proliferation, clonogenic growth, migration, invasion, and *in-vivo* tumor burden, indicating that LAP3 is not merely correlative but can modulate malignant behavior under the conditions tested. As a leucine aminopeptidase shaping intracellular peptide and amino-acid pools, LAP3 plausibly couples nutrient availability and proteostasis to signaling, antigen handling, and stress responses.

These features suggest translational opportunities. LAP3 delineates spatially coherent metabolic niches that coincide with checkpoint and antigen-presentation activity; combined with chemokine and checkpoint panels, it may stratify patients into metabolism-immune states with differing propensities for response to immunotherapy or to metabolic interventions. Therapeutically, the data motivate trials that pair metabolic modulation with PD-1/PD-L1 blockade, using spatial LAP3 as a companion biomarker.

Future work should deploy cell-type–specific CRISPR perturbations in epithelial and macrophage compartments, couple them with metabolomics, stable-isotope flux tracing, and phospho-signaling readouts, and use co-culture and spatial multi-omics to visualize how LAP3-high macrophages organize niches in real time. Preclinical studies that modulate the LAP3 axis or upstream transporters/peptidases in combination with immune checkpoint therapy will be crucial. Altogether, the data support a metabolism–communication nexus centered on LAP3 that shapes tissue structure and clinical behavior, offering a concrete framework for biomarker development and rational metabolism-immune combination strategies.

## 5 Conclusions

This study identifies LAP3 as a central node in a metabolism–immunity axis in NSCLC, enriched in tumor cells and a distinct LAP3-high macrophage subset, where it coordinates chemokine, cytokine, and adhesion signaling at epithelial–myeloid interfaces. High LAP3 expression defines an NSCLC subtype characterized by concurrent metabolic activation and immune checkpoint upregulation—a “metabolic-immunosuppressive” phenotype—and suppresses tumor aggressiveness *in vitro* and *in vivo*. Key limitations include incomplete mechanistic insight into how LAP3 enzymatic activity directly modulates immunity, reliance on analysis data and AI models, and uncharacterized dynamics of LAP3 during therapy.

## Data Availability

The original contributions presented in the study are included in the article/**Supplementary Material**. Further inquiries can be directed to the corresponding authors.

## Supplementary material

Supplementary Figure 1 The LAP3 characteristic gene induces the progression of the disease. (A) Expression status of LAP3 in all types of cancers. **(B)** Expression of LAP3 in TCGA-LUAD. **(C)** Mutation and expression status of LAP3.
