# Supplementary figures and images for: Decoding the role of macrophage LAP3 in lung cancer – integration of single-cell technologies and machine learning reveals an orchestrating immunometabolic circuit at the tumor-epithelial interface
Source: Front Immunol. 2026 Feb 27;17:1749190. doi: 10.3389/fimmu.2026.1749190 (PMC12982070; doi:10.3389/fimmu.2026.1749190)

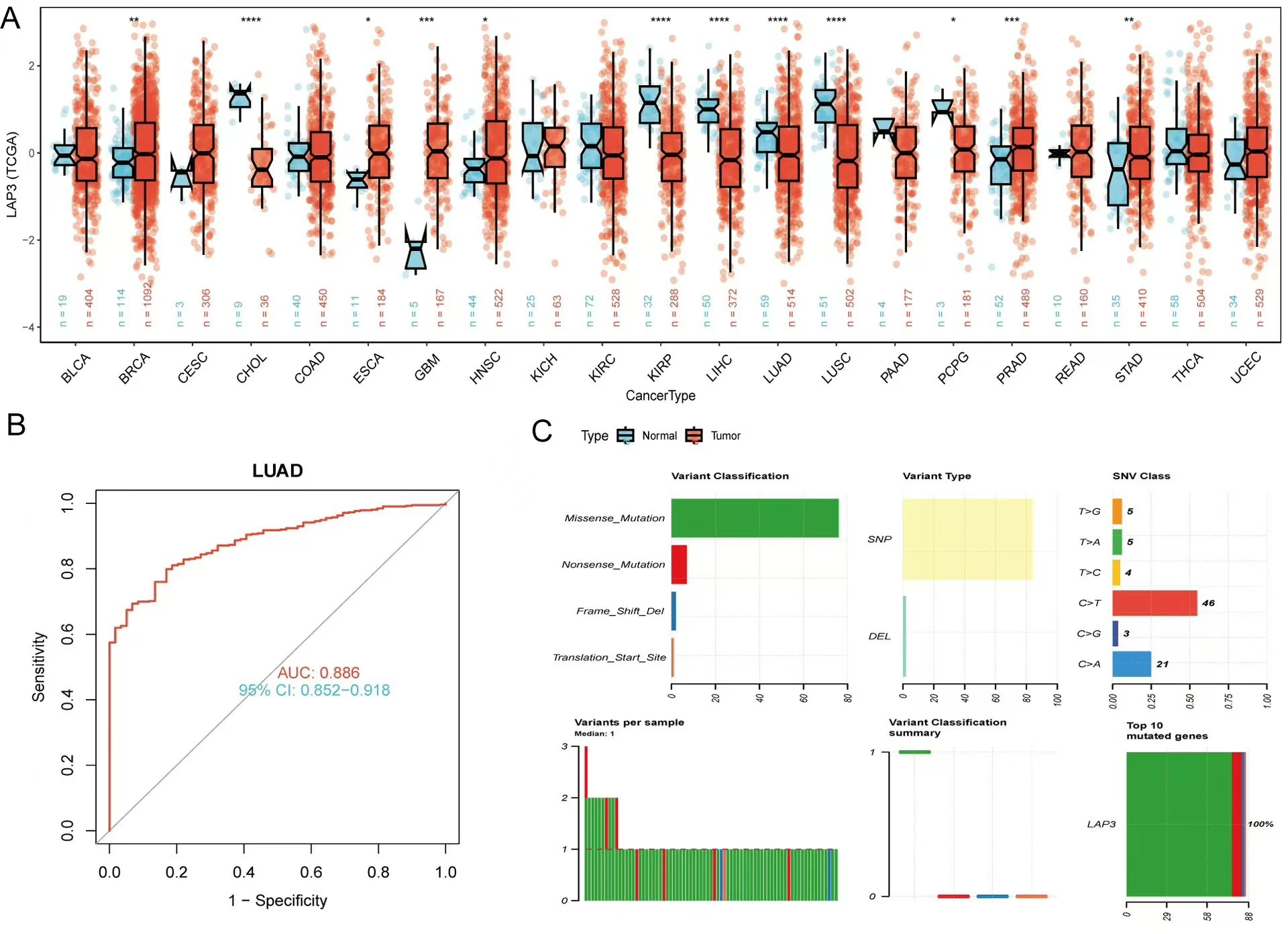

Supplement: Supplementary Figure 1 — The LAP3 characteristic gene induces the progression of the disease. (A) Expression status of LAP3 in all types of cancers. (B) Expression of LAP3 in TCGA-LUAD. (C) Mutation and expression status of LAP3. [file Image1.tif]
